# Toward Understanding Bacterial Ice Nucleation

**DOI:** 10.1021/acs.jpcb.1c09342

**Published:** 2022-01-27

**Authors:** Max Lukas, Ralph Schwidetzky, Rosemary J. Eufemio, Mischa Bonn, Konrad Meister

**Affiliations:** † Max Planck Institute for Polymer Research, 55128 Mainz, Germany; ‡ University of Alaska Southeast, Juneau, Alaska 99801, United States

## Abstract

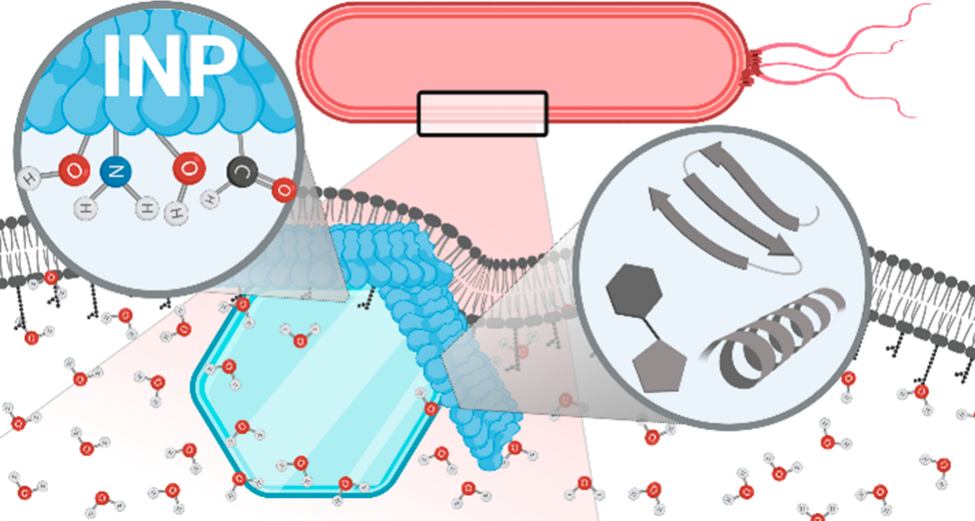

Bacterial ice nucleators (INs) are among the most effective ice nucleators known and are relevant for freezing processes in agriculture, the atmosphere, and the biosphere. Their ability to facilitate ice formation is due to specialized ice-nucleating proteins (INPs) anchored to the outer bacterial cell membrane, enabling the crystallization of water at temperatures up to −2 °C. In this Perspective, we highlight the importance of functional aggregation of INPs for the exceptionally high ice nucleation activity of bacterial ice nucleators. We emphasize that the bacterial cell membrane, as well as environmental conditions, is crucial for a precise functional INP aggregation. Interdisciplinary approaches combining high-throughput droplet freezing assays with advanced physicochemical tools and protein biochemistry are needed to link changes in protein structure or protein–water interactions with changes on the functional level.

## Introduction

Freezing processes in the atmosphere have a significant influence on the formation of clouds, on precipitation patterns, and on Earth’s energy balance.^1,2^ Homogeneous ice nucleation at a given temperature requires a certain number of ice-like water molecules. The precise homogeneous nucleation temperature depends on droplet volume, pressure, and the water activity in the presence of potential solutes.^3^ Pure water can be supercooled to temperatures as low as −38 °C.^3,4^ Above the homogeneous freezing point, ice crystal formation is triggered by particles that serve as heterogeneous ice nucleators (INs). Numerous INs have been identified and their ice nucleation efficiencies are typically characterized using droplet freezing assays.^5−9^ In such assays, a large number of droplets containing a well-defined concentration of INs is gradually cooled down and the fraction of frozen droplets as a function of temperature is recorded. The temperature at which half of the droplets are frozen, *T*_50_, provides a direct measure for the efficacy of the IN. While mineral dust-based INs (e.g., feldspars, silicates, clay minerals) play a major role in the atmosphere owing to their ubiquity, the ice nucleation efficiency of biological INs derived from bacteria, fungi, lichen, or plants is much higher.^5^ Despite its significance and the acceleration of research in this field in recent years, several questions on the molecular-level mechanisms of heterogeneous ice nucleation remain unanswered. This makes it difficult to predict the decisive properties of efficient INs and their role in the environment. Understanding such molecular-level mechanisms could point to novel ways of triggering ice nucleation, desirable not only for artificial snow, for instance, but also for new artificial anti-icing surfaces.^10−12^

Ice-nucleation activity in bacteria was first discovered in *Pseudomonas* in the 1970s.^13,14^ Subsequently, several other ice-nucleating bacteria belonging to species in the *Pseudomonadaceae*, *Enterobacteriaceae*, *Xanthomonadaceae*, and *Lysinibacillus* families have been identified.^15−17^ The best-characterized bacterial INs are *Pseudomonas syringae*, which enable ice nucleation at temperatures at −2 °C. The ability of bacteria to facilitate ice formation is attributed to specialized proteins anchored to the outer bacterial cell membrane. As a plant pathogen, *P. syringae* causes frost injury to the plant tissue by increasing the nucleation temperature of water, which enables access to nutrients.^9^ Moreover, like many other ice-nucleating microbes, *P. syringae* was identified in ice, hail, and snow, indicating that they might contribute to freezing processes in the atmosphere.^5,18^ The unique standing of *P. syringae* as a source of exceptional bacterial INs is further emphasized by its commercialization as Snomax. This artificial snowmaking product consists of extracts of sterilized *P. syringae*.

**Figure 1:**
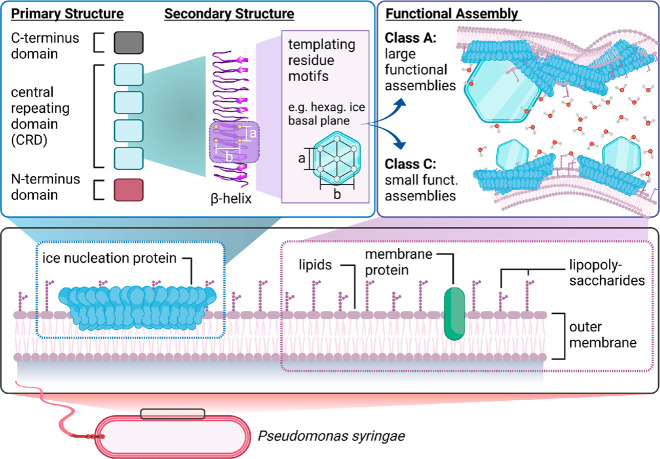
Overview of the proposed structure and working mechanism of bacterial ice nucleation proteins anchored to the outer cell membrane of *P. syringae*. The INP consists of an N-terminal, a C-terminal, and a central repeating domain. Their general function is to order water molecules into an “ice-like” arrangement to nucleate ice formation. This process is facilitated when INPs assemble into larger aggregates.

The biomolecules responsible for bacterial ice nucleation are large ice nucleation proteins (INPs) anchored to the outer membranes of the bacterial cells, as schematically shown in Figure 1. The principal function of the INPs is to order water molecules into an “ice-like” arrangement, thereby facilitating the kinetically hindered phase transition.^19−25^

The amino acid sequence of the INPs of *P. syringae* has been deduced and is widely used to model its structure as shown in Figure 1.^25−28^ The INP consists of three domains: (1) a central repeating domain (CRD) comprising ∼81% of the total sequence, (2) an N-terminal domain comprising ∼15% of the sequence, and (3) a C-terminal unique domain (∼4%). The CRD has been proposed to contain the ice nucleation site of the INPs, and molecular simulations have shown that the active site consists of similarly effective hydrophobic TxT and hydrophilic ExSxT amino acid motifs.^29^

The large size and embedment into the membrane still hamper experimental attempts to solve the three-dimensional structure and associated molecular-level details of the INPs. In contrast, the structures of antifreeze proteins (AFPs) containing similar TxT motifs have been solved, oftentimes revealing β-solenoid folds.^19,24,30^ A β-helical motif has also been used to model the structure of bacterial INPs,^31^ on the basis of the idea that AFPs and INPs share similar folds and ice-binding motifs.^20,29,32^

A central enigma of bacterial ice nucleation arises from the broad distribution of threshold nucleation temperatures ranging from −2 to −12 °C. This is reflected in freezing assays that show not one single *T*_50_ but a wider range of nucleation temperatures. On the basis of extensive freezing assays of *P. syringae* for different concentrations, three distinct classes of INs have been proposed.^33,34^

Govindarajan and Lindow showed that the largest structures of INs reach the highest threshold temperature, i.e., nucleate ice most efficiently.^35^ Southworth et al. revealed a nonlinear relationship between ice nucleation activity and the concentration of INPs in bacterial cells.^36^ Together, those findings indicate that the different activation temperatures can be explained by aggregation of INPs, thereby varying the accumulated size of the ice nucleation site. These protein aggregates provide another example of how protein aggregation can have beneficial effects to cellular systems.^37^ Simulations have addressed the role of size and aggregation of the proteins on the freezing temperature and provided quantitative predictions of the ice nucleation temperature vs the number of proteins in the aggregates, as well as to the distance between the monomers in the aggregates.^38^ On the basis of freezing assays, the predominant and least efficient fraction of bacterial INs active at ∼−7 °C, *Class C*, has been attributed to small aggregates of INPs (5–10 INPs^38^).^33^ The most active *Class A* INs are active at temperatures up to ∼−2 °C and consist of the largest aggregates of the INPs (>30 INPs^38^).^33^ Class B INs are rarely observed and responsible for freezing between ∼−5 and ∼−7 °C. Aggregation of the INPs in the cell membrane was described in several studies and it has further been suggested that the membrane plays a major role in enabling the highly active Class A INs.^36,39,40^

## Methods

Progress in unraveling the mechanism underlying bacterial ice nucleation requires advanced physicochemical methods and interdisciplinary approaches. Essential for any investigation of INs are droplet freezing assays. High-throughput assays, like the Twin-plate Ice Nucleation Assay (TINA), now enable the simultaneous measurement of complete dilution series (typically 0.1 mg/mL to 1 ng/mL) with robust statistics, enabling the cumulative representation of the complete range of present INs.^41^ Observations at the functional level can be accompanied by molecular-scale investigations using spectroscopic tools. Circular dichroism and infrared spectroscopy provide information on the secondary structure, while surface-specific vibrational sum-frequency generation spectroscopy (SFG) is a powerful tool to investigate the molecular-level details of the interface of bacterial INPs and water.^42−46^ The biophysical and spectroscopic investigations are further highly dependent on sample quality. Recent progress in ice-affinity purification methods now allows for isolating ice-binding proteins directly from natural sources and with high purity.^44,47−49^ In the studies presented here, we utilized inactivated extracts from *P. syringae*, commercially available under the product name Snomax (Snomax Int.).

## Results and Discussion

**Figure 2:**
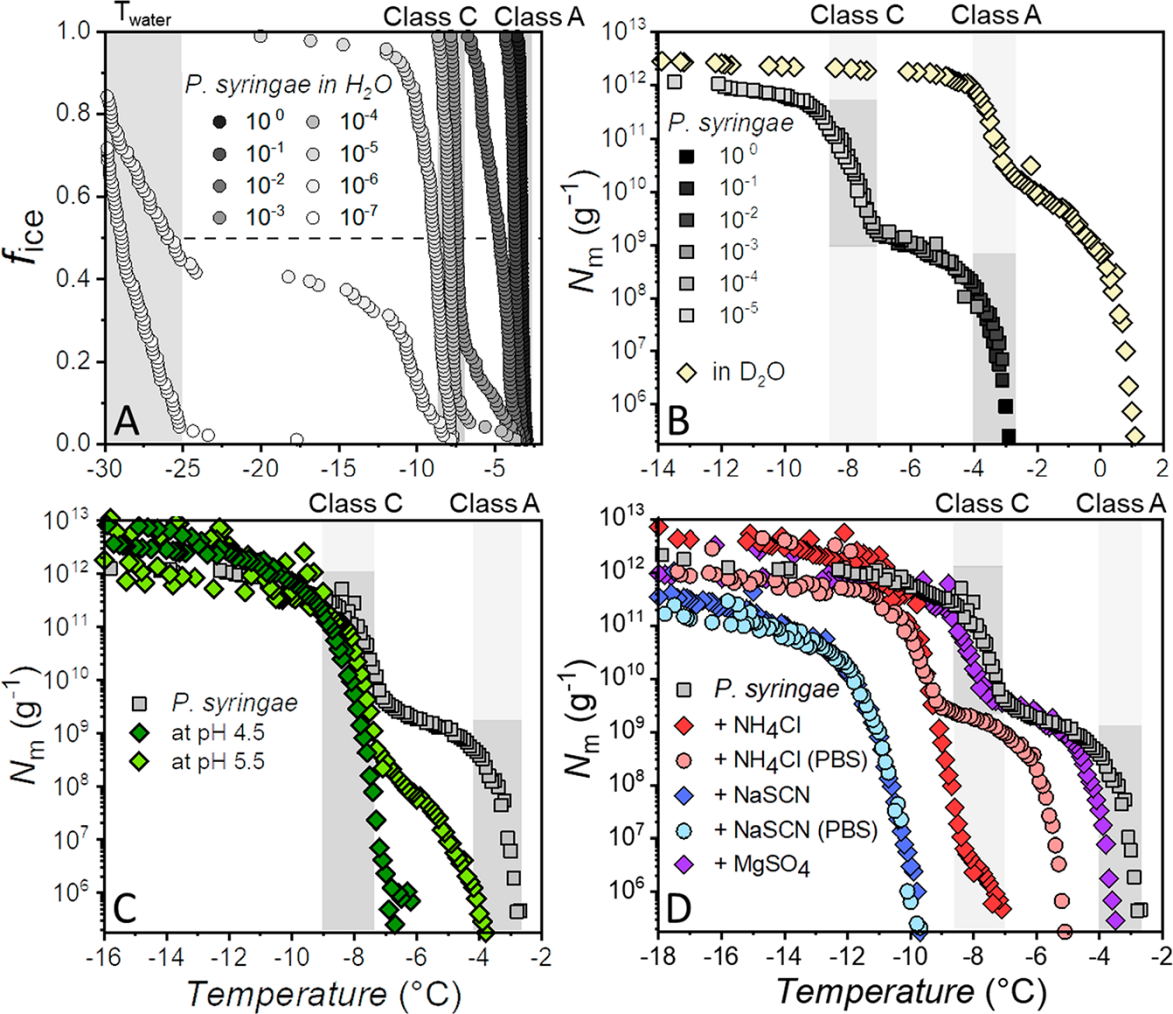
Freezing spectra of aqueous solutions of Snomax, containing bacterial ice nucleators from *P. syringae*. (A) Fraction of frozen droplets (*f*_ice_) vs temperature for the dilution series of a *P. syringae* measurement in pure water. (B) Cumulative freezing spectra of *P. syringae* in pure H_2_O and D_2_O. (C) Freezing spectra of *P. syringae* at pH 6.2 (gray), 5.5 (light green), and 4.5 (dark green), adapted with permission from ref (42). Copyright 2020 American Chemical Society. (D) Freezing spectra of *P. syringae* in pure water and in the presence of 0.5 mol/kg MgSO_4_ (purple), NaSCN (blue), NH_4_Cl (red) in water and of NaSCN (light blue), NH_4_Cl (light red) in PBS buffer adapted with permission from ref (46). Copyright 2021 Wiley-VCH. The temperature ranges of Class A and Class C are highlighted in gray and correspond to measurements of *P. syringae* in pure water.

Figure 2 shows freezing spectra of bacterial ice nucleators from *P. syringae* under different environmental conditions. All cumulative freezing spectra are composed of measurements of a 10-fold dilution series. The fraction of frozen droplets (*f*_ice_) measurements shown in Figure 2A correspond to the spectra of *P. syringae* INs in pure water (gray curves) in Figure 2B. The cumulative IN concentration (*N*_m_) is calculated using Vali’s equation^50^ and represents the number of ice nucleators per unit weight that are active above a certain temperature. The two strong increases at ∼−3 and ∼−7.5 °C correspond to the large aggregates (Class A INs) and the smaller aggregates (Class C INs), respectively. The two increases are followed by plateaus, which indicate that fewer INs are active in those temperature ranges.^51^

Figure 2B shows the results of ice nucleation measurements of the bacterial INs in deuterated water (D_2_O). The freezing spectrum is shifted ∼+4 °C, which is consistent with the expected shift of +3.82 °C based on the higher melting point of D_2_O. Turner et al. previously described a third intermediate Class B of INs, active at around −5 °C, and that examining the effects of substituting D_2_O for H_2_O allows for differentiation of the different classes on the basis of their isotope-induced shifts in nucleation threshold.^33^ As apparent from Figure 2B, the freezing spectra do not show an additional increase assignable to a third class of INs. However, differences in the freezing curves of *P. syringae* in H_2_O and D_2_O do occur. Measurements in D_2_O show a larger number of Class A INs and fewer Class C INs. We explain the observed differences with higher rigidities of INPs in D_2_O and fewer structural fluctuations of the INP aggregates due to the stronger intramolecular D-bonds.^52^

Several studies have reported that pH changes of the aqueous solution or the addition of cosolutes affects the Class A INs differently than Class C.^33,34^Figure 2C shows cumulative freezing curves of *P. syringae* as a function of pH.^42^ Upon lowering the solution pH, the first rise at ∼−3 °C (Class A) gradually decreases and shifts to lower temperatures while the fraction of INs active at ∼−7.5 °C (Class C) increases. There seems to be a clear interconversion of Class A species into Class C species with decreasing pH. At a pH of ∼4.5, we observe that only Class C INs remain active.

**Figure 3:**
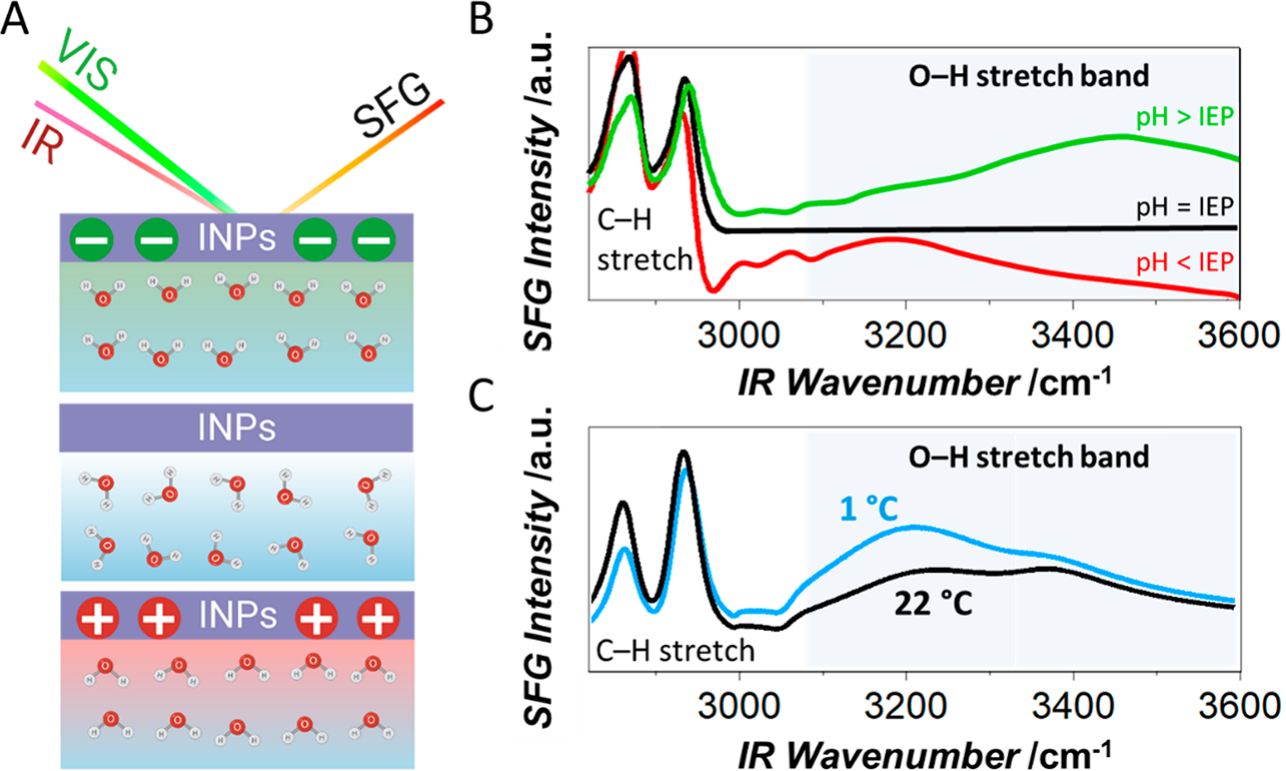
Sum-frequency generation (SFG) spectroscopy of bacterial INPs at the surface of aqueous solutions. (A) The incident IR and VIS beams generate a surface-specific SFG signal from the vibrational resonances. The illustration shows the alignment of interfacial water molecules in the case of a negative net charge as found at the natural pH of ∼6.2, in the case of zero net charge at the isoelectric point (IEP) ∼ 4.2, and the opposite alignment in the case of a positive net charge at pH values below the IEP. (B) Corresponding SFG spectra. The O–H band intensity is close to zero at the IEP and increases with the charge-induced alignment of the water molecules. The flip of the molecules’ orientations causes a frequency shift of the O–H stretch band. (C) Temperature-dependent SFG spectra of the O–H stretch band of interfacial H_2_O molecules and the C–H stretch vibrations. The intensity of the O–H stretch band, and therefore the interfacial water alignment, is significantly higher at low temperatures.^42,44^

By using interface-specific SFG vibrational spectroscopy as a tool for the determination of the isoelectric point of the bacteria, a possible explanation for this puzzling disappearance of Class A aggregates could be obtained.^42^ In SFG spectroscopy, a broadband infrared (IR) beam is used to probe the molecular vibrations in a given frequency region (Figure 3A). The IR beam is combined with a visible beam (VIS) at the sample surface to generate light of the sum-frequency of the two incident fields. This second-order nonlinear process is bulk-forbidden in isotropic media and only ensembles of molecules with a net orientation, e.g., at an interface, generate a detectable signal.

The SFG signal intensity in the O–H stretch region increases with the alignment of the water molecules’ dipoles, as, e.g., induced by the net charge of a protein film on the surface (Figure 3A). Consequently, SFG can be used to determine the isoelectric points (IEPs) of proteins by monitoring the O–H stretch signal (Figure 3B).^53−56^

The IEP of the *P. syringae* determined with SFG was found to be ∼4.2, which coincides with the pH at which the Class A INs are completely absent. Apparently, the repulsive forces caused by the net negative charge of the INPs are crucial for the precise alignment of the Class A aggregates, which rely on sub-Ångstrom control over the distances of the single INPs’ active sites.^38^

A combination of TINA and SFG experiments further revealed ion-specific effects on *P. syringae* INs that follow the Hofmeister series.^46^Figure 2D shows bacterial freezing spectra in the presence of different salts. NaCl (not shown) was found not to affect the bacterial freezing spectrum except a shift of around −2 °C caused by colligative melting point depression.^57^ In contrast, freezing spectra of bacterial solutions containing NH_4_Cl, MgSO_4_, and NaSCN, show ion-specific effects. NH_4_Cl causes the first rise at −3 °C to shift to ∼−7.5 °C, close to the second rise, now found at ∼−9 °C. Interestingly, when freezing spectra of buffered and unbuffered solutions containing NH_4_Cl are compared, this effect is solely explainable by salt-induced solution pH changes. In the presence of NaSCN, only a single increase at ∼−11.5 °C remains, indicating a complete loss of Class A and a partial inhibition of Class C INs. The effect is similar for the buffered solution, excluding a pH effect. In the presence of MgSO_4_, no inhibition is observed. In fact, after correcting for the colligative freezing point depression, the freezing curve is shifted to warmer temperatures, suggesting enhanced ice nucleation efficiency. Comprehensive studies of 16 salts showed that their effects on the INP-mediated freezing temperatures follow the trend of the anions in the Hofmeister series. Weakly hydrated ions, such as thiocyanate, lower the threshold temperatures while more strongly hydrated ions, such as sulfate, have no effect or can apparently facilitate ice nucleation.

SFG experiments revealed that although the ionic strengths and counterions are identical, the salts have different efficiencies in screening the net charge of the bacteria. Weakly hydrated anions decrease the SFG intensity less than strongly hydrated ions. Supported by MD simulations, we explained these results in terms of two effects: Compared to strongly hydrated anions, the weakly hydrated anions preferentially adsorb to the bacterial surfaces, which renders the bacterial surfaces more negative and increases the order of the interfacial water molecules. Additionally, the ions might induce changes in the INP conformation and thereby affect the charge distribution.

The high sensitivity of SFG to the ordering of interfacial water molecules raises the question of whether specific ice-like ordering of water in contact with INPs can be observed close to their biologically relevant working temperature. Pandey et al. reported SFG experiments of *P. syringae* (Snomax) in D_2_O at room temperature and 1 °C above the melting point and showed that the SFG signal in the O–D stretch region is increased and red-shifted at low temperature, indicating an increase in the alignment of the water molecules.^43^ Shortened INPs with low ice nucleation activity expressed in *E. coli* showed a similar effect, and the observation was attributed to an activation of INPs at lower temperature and the ability to order water, which increases close to the respective freezing temperature.^45^ While providing much needed experimental insights into the INP/water interface, these studies and interpretations must be taken with a caveat, given that more recently it has been shown that water ordering at lower temperatures observed with SFG (Figure 3C) are identical for active INPs and heat-denatured INPs that have completely lost their ice nucleation activity.

## Conclusions

**Figure 4:**
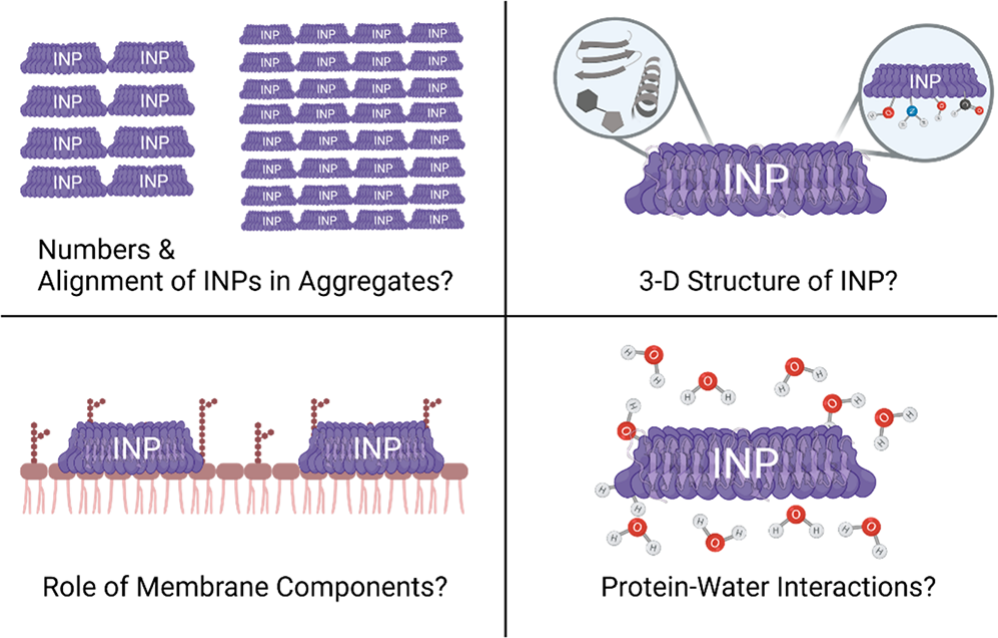
Overview of open questions toward understanding the molecular-level mechanisms of bacterial ice nucleation.

From our recent studies, we conclude that the outstanding ice nucleation efficiency of bacteria can only be understood in the study of the natural, functional aggregation of the protein. It is evident that a membrane-associated mechanism is responsible for the formation of large Class A aggregates, which are responsible for the exceptionally high freezing temperatures (∼−2 °C) close to water’s melting temperature. The process of bacterial ice nucleation at warm temperatures requires an appropriate pH value and intact INP structures.^44^ Moreover, the activity of both classes of bacterial INs is strongly influenced by specific interactions with ions. These interactions are highly relevant to correctly predict the ice nucleation efficiency of bacterial INs under natural conditions (e.g., in the atmosphere). The important role of functional aggregation is further underlined by simulation studies, which have shown that not only Class A but also the smaller Class C INs active at around −7.5 °C are a product of functional aggregation of the proteins and merging of their active sites.^38^ Our studies of purified INPs from *P. syringae* have underlined the importance of the membrane for the formation of Class A aggregates,^44^ emphasizing its essential role for the ice nucleation activity. We hypothesize that the formation of Class C aggregates might have another molecular mechanism than the membrane-associated mechanism responsible for forming the larger Class A aggregates. Clarification of whether the membrane’s role lies merely in providing a matrix or whether it is part of the active ice nucleation site is another critical step for unraveling the molecular origin of bacterial ice nucleation.^58^ In addition to unsolved questions regarding the 3D structure of the INP monomer and the interfacial structure of water at the functional site of the INP, information on the precise numbers of INPs in the aggregates, their alignment, and which interactions (e.g., hydrophobic effect, ionic interactions) drive the aggregation is needed (Figure 4).

Understanding the molecular-level processes driving bacterial ice nucleation may provide further insights into the role of biological INs in the environment. Answering these questions will likely also enable the community to unravel how nature precisely aligns INPs to be the most efficient ice nucleators known and illuminate how this strategy can be copied for new freezing products and technologies.
